# From initial trust to critical reconstruction: upper secondary students’ engagement with generative AI in science learning

**DOI:** 10.3389/fpsyg.2026.1830646

**Published:** 2026-05-22

**Authors:** Chun Zhou

**Affiliations:** School of Arts, Sichuan Preschool Educators College, Mianyang, China

**Keywords:** generative artificial intelligence, science learning, scientific literacy, trust calibration, upper secondary students

## Abstract

**Introduction:**

This study examines how upper secondary students engage with generative artificial intelligence (GenAI) in science learning, focusing on how they move from initial trust to critical reconstruction of AI-generated content.

**Methods:**

Using a constructivist grounded theory design, the study drew on semi-structured interviews with 21 students aged 15-18 from two upper secondary schools in Southwest China.

**Results:**

The findings show a dynamic process shaped by task demands, prior knowledge, time pressure, and teacher norms. Students often began with efficiency-driven use and provisional trust in fluent, authoritative responses. They then evaluated outputs through internal consistency checking, external corroboration, and growing awareness of GenAI’s limitations. Based on these judgements, students refined prompts, selectively reconstructed content, or abandoned GenAI when outputs were unreliable.

**Discussion:**

The study argues that GenAI use in science learning is best understood as a form of regulated epistemic engagement.

## Introduction

Generative artificial intelligence (GenAI), especially large language models such as ChatGPT, is rapidly entering students’ everyday learning practices. In educational settings, these tools can generate explanations, summaries, examples, and extended responses on demand and can become part of students’ day-to-day learning routines. Recent meta-analyses and systematic reviews suggest that GenAI can support student learning, engagement, and motivation under appropriate conditions, but they also show that its effects vary considerably across tasks, disciplines, and instructional arrangements (Deng et al., 2025; Heung and Chiu, 2025; Lo et al., 2024). Reported use alone therefore tells us little about educational value. In science learning, the key issue is how students interpret, check, and act on GenAI outputs in concrete tasks.

This issue is especially consequential in science learning. Students now increasingly turn to GenAI for information seeking, clarifying complex concepts, summarizing content, answering study-related questions, and supporting essays or other written assignments (Morell-Mengual et al., 2025; Blahopoulou and Ortiz-Bonnin, 2025). However, science learning is not reducible to receiving plausible answers. It requires students to evaluate evidence, distinguish explanation from assertion, coordinate claims with disciplinary reasoning, and judge whether knowledge claims are sufficiently warranted. In this context, the convenience and fluency of GenAI are double-edged. The same features that make GenAI attractive—speed, coherence, and an authoritative tone—may also encourage learners to confuse plausibility with validity. Recent educational analyses have therefore warned that GenAI can become epistemically risky when learners rely on it uncritically or treat machine-generated responses as trustworthy by default (Farrokhnia et al., 2024).

Although research on GenAI in education is expanding rapidly, three limitations remain particularly salient. First, much of the existing literature is concentrated in higher education. Reviews of AI and GenAI in education consistently show that university settings dominate the evidence base, whereas school-level and especially upper secondary subject learning remain comparatively underexamined (Deng et al., 2025; Lee and Kwon, 2024). This imbalance matters because upper secondary science learning is shaped by distinctive epistemic and instructional conditions, including stronger curricular accountability, high-stakes assessment, and tighter alignment with formal disciplinary norms. Findings from higher education therefore cannot simply be transferred to school science contexts.

Second, a large proportion of current studies focus on adoption, attitudes, acceptance, or general usage patterns. This work has been valuable in showing that students often perceive ChatGPT and related tools as useful for efficiency, clarification, drafting, and idea generation, while also expressing concerns about accuracy, dependency, and policy uncertainty (Acosta-Enriquez et al., 2024; Baek et al., 2024; Polyportis and Pahos, 2025; Stöhr et al., 2024). Yet such research remains limited in its ability to explain what happens after a learner receives a GenAI output. In science learning, the central question is how students decide whether a specific explanation, answer, or line of reasoning is trustworthy enough to use, revise, or reject.

Third, the process dimension of students’ GenAI engagement remains insufficiently theorized in school science education. Existing studies suggest that learners’ reliance on GenAI is heterogeneous and task-dependent rather than uniform (Stojanov et al., 2024). School-based work has also shown that ChatGPT can support science learning and self-regulated learning when its use is scaffolded pedagogically (Ng et al., 2024a). Science education scholarship has also raised epistemic questions about the credibility, limits, and knowledge consequences of AI-generated representations (Cheung et al., 2025; Erduran and Levrini, 2024). However, little is known about how upper secondary students, in ordinary and relatively unstructured science learning, move from initial exposure to a GenAI response toward later evaluative and behavioral decisions.

This gap can be more precisely understood by bringing together three lines of scholarship. Research on trust in artificial intelligence emphasizes appropriate reliance calibrated to system capability, task requirements, and situational risk (Glikson and Woolley, 2020; Lee and See, 2004). Self-regulated learning theory similarly suggests that productive tool use depends on learners’ planning, monitoring, and reflection (Zimmerman, 2000). In addition, science education has long stressed that scientific literacy involves evaluating evidence, reasoning about expertise, and engaging critically with knowledge claims (Bromme and Goldman, 2014; Feinstein, 2011). Taken together, these perspectives support examining GenAI use in science learning as a dynamic process of epistemic judgment and regulatory action.

The present study addresses this problem by investigating how upper secondary students engage with GenAI outputs in science learning. Rather than treating GenAI use as a static matter of acceptance or frequency, the study conceptualizes it as a process extending from initial trust to critical reconstruction. Specifically, it explores how students initiate interaction with GenAI, how they evaluate the reliability and task-appropriateness of AI-generated scientific content, and how those evaluative judgments shape subsequent actions such as selective integration, revision, and rejection. To address these aims, the study examines three interrelated research questions: (1) How do upper secondary school students initiate interaction with GenAI in science learning tasks and form an initial acceptance orientation? (2) How do they evaluate the reliability and task-appropriateness of GenAI-generated scientific content? and (3) What strategies do they adopt, based on their evaluative judgments, to process, integrate, revise, or reject GenAI-generated content in science learning?

## Literature review

### Generative AI in education and science learning

Research on generative artificial intelligence (GenAI) in education has expanded rapidly, with a growing body of evidence suggesting that tools such as ChatGPT can support students’ learning performance, engagement, and motivation under appropriate instructional conditions (Deng et al., 2025; Heung and Chiu, 2025; Lo et al., 2024). At the same time, recent reviews caution against treating GenAI as a neutral efficiency tool. Its educational value depends on access to the technology and on how learners interact with, interpret, and regulate AI-generated outputs during disciplinary learning (Farrokhnia et al., 2024; Feuerriegel et al., 2024).

A notable feature of the existing literature is the dominance of higher education contexts. Many empirical studies focus on university students’ attitudes, intentions, or self-reported practices, showing that learners often value GenAI for brainstorming, clarification, drafting, and time-saving support (Acosta-Enriquez et al., 2024; Baek et al., 2024; Stöhr et al., 2024). Other studies suggest that students’ adoption of ChatGPT is shaped by trust, anthropomorphism, design novelty, and institutional policy contexts (Polyportis and Pahos, 2025). This line of work has been useful in identifying broad acceptance patterns, but it primarily captures whether students are willing to use GenAI rather than how they actually process its outputs during learning.

This limitation is particularly salient in school education. Although K–12 AI education has become an increasingly active field, much of the research has concentrated on AI literacy, curriculum design, and general classroom applications rather than students’ engagement with GenAI in subject-specific learning tasks (Lee and Kwon, 2024). In science education, the number of empirical studies remains comparatively small. Existing work suggests that ChatGPT can support science learning when its use is scaffolded and embedded in instructional design (Ng et al., 2024a), and emerging evidence from school and STEM settings indicates that students often perceive GenAI as helpful for explanation, clarification, and task support (Conrad and Nuebel, 2025; Valeri et al., 2025). However, current research still provides limited insight into how upper secondary students engage with GenAI outputs in the course of authentic science learning, particularly when they must independently decide whether an output should be accepted, checked, revised, integrated, or discarded.

This gap is consequential because science learning places distinctive demands on learners. In contrast to routine information retrieval, effective science learning requires the interpretation of concepts, the evaluation of evidence, and the coordination of claims with disciplinary reasoning. A plausible answer is not necessarily a trustworthy one, and a fluent explanation is not necessarily a scientifically sound one. The key educational issue is how students manage GenAI in contexts where epistemic quality matters. This calls for process-oriented analyses of students’ real engagement with AI-generated scientific content.

### Trust calibration, self-regulated learning, and evaluative engagement with GenAI

A useful conceptual lens for understanding students’ GenAI use is research on trust in artificial intelligence. In human–AI interaction research, trust is generally understood as appropriate reliance calibrated to the capabilities, limitations, and context of system use (Glikson and Woolley, 2020; Lee and See, 2004). In educational settings, this perspective is especially important because GenAI systems often produce outputs that are fluent, coherent, and authoritative in tone, even when they contain inaccuracies, oversimplifications, or fabricated information. As a result, learners may be inclined either to over-trust the system because of its apparent competence or to reject it altogether because of known risks. Neither response reflects calibrated use.

From an educational perspective, calibrated trust is closely related to self-regulated learning. Self-regulated learning theory conceptualizes effective learning as a cyclical process involving forethought, monitoring, control, and reflection (Zimmerman, 2000). Applied to GenAI, this suggests that productive engagement requires more than initial adoption. Learners must set a purpose for use, monitor the adequacy of AI-generated responses, compare them with task demands and prior knowledge, and decide how to act on the basis of those judgments. In this sense, GenAI becomes part of the learner’s regulatory environment rather than a simple external aid.

Recent educational research supports this view. Ng et al. (2024a) found that ChatGPT could enhance both science learning and self-regulated learning when students’ interactions with the tool were pedagogically scaffolded. Other studies indicate that the educational quality of GenAI use depends on the depth of interaction it elicits. For example, Suriano et al. (2025) reported that student interaction with ChatGPT could promote more complex critical thinking under suitable conditions, while Urban et al. (2025) showed that students’ epistemic beliefs and metacognitive accuracy influenced whether they integrated ChatGPT-generated content critically or too readily in academic writing tasks. These findings suggest that learner differences in evaluation and regulation are central to understanding GenAI use.

At the same time, much of the current literature still treats trust, AI literacy, or engagement as relatively stable dispositions rather than as unfolding judgment processes. AI literacy frameworks, for instance, usefully specify learners’ readiness to work with AI across cognitive, behavioral, affective, and ethical dimensions (Ng et al., 2024b), but such frameworks do not by themselves explain what students do when they encounter a concrete GenAI response that may be partially useful, partially misleading, or misaligned with disciplinary expectations. Similarly, latent profile studies can identify distinct reliance patterns among students (Stojanov et al., 2024), yet they provide limited insight into the micro-level sequence through which reliance is enacted and revised in task-specific situations.

For this reason, it is important to conceptualize students’ engagement with GenAI as an evaluative process. The key educational question is how students’ trust is formed, challenged, recalibrated, and translated into subsequent action. In science learning, such action may include corroborating content with textbooks or class notes, checking claims against scientific principles, revising inaccurate explanations, selectively integrating useful parts, or abandoning outputs judged to be unreliable. A process-oriented account of trust calibration is therefore essential for explaining how GenAI use becomes either a support for learning or a source of epistemic vulnerability.

### Epistemic demands of science learning and the need for a process-oriented perspective

The need for a process-oriented perspective becomes even clearer when GenAI is situated within the epistemic demands of science learning. Science education has long emphasized that scientific literacy involves more than mastering factual knowledge; it includes the capacity to interpret evidence, evaluate explanations, and reason about expertise and the production of knowledge in society (Feinstein, 2011). In practice, however, learners cannot verify all scientific claims directly. They must often rely on expert-generated or institutionally authorized information while simultaneously maintaining a degree of critical vigilance toward sources and representations of knowledge (Bromme and Goldman, 2014).

This issue becomes more complex in AI-mediated environments. GenAI outputs can simulate expertise through technical vocabulary, coherent prose, and confident formulations, but they typically do not make the epistemic basis of their statements transparent. Learners therefore need to evaluate content and decide whether it is reliable enough for the task at hand. Recent work on epistemic vigilance is especially relevant here. Bielik and Krell (2025) argue that epistemic vigilance includes attention to the credibility of information, the reliability of sources, and the receiver’s own cognitive limitations and biases. In the context of GenAI, this means that students must evaluate both the output and their own readiness to accept it too quickly, especially when it appears convincing.

Research on online information evaluation reinforces this point. Studies on lateral reading and digital source evaluation have shown that effective judgment requires learners to corroborate information, inspect credibility cues, and move beyond surface plausibility (Breakstone et al., 2021; Wineburg et al., 2022). These insights are highly pertinent to GenAI-supported science learning, where plausible but incorrect explanations may be particularly difficult for students to detect. Empirical work in science education already suggests that this is a real challenge. Cooper (2023) found that ChatGPT could generate scientifically relevant responses, but also produced inaccuracies, oversimplified explanations, and plausible-sounding formulations whose epistemic basis remained unclear for educational use. Cheung et al. (2025) further concluded from a systematic review that epistemic insights remain underdeveloped in science education research on AI.

Recent scholarship also indicates that the issue is not confined to classroom tool use but relates to broader changes in the knowledge landscape of science itself. Erduran and Levrini (2024) argue that AI is reshaping scientific practices and therefore poses new questions for science education about how knowledge is generated, communicated, and trusted. Under these conditions, helping students use GenAI efficiently is insufficient. What matters is whether students can engage with AI-generated scientific content in ways that preserve disciplinary rigor and support meaningful learning.

Taken together, the literature points to a clear need for research that examines upper secondary students’ engagement with GenAI in science learning as a dynamic process rather than a static attitude or generalized usage preference. Existing studies help explain why students adopt GenAI and what benefits or concerns they report, but they do not yet adequately capture how learners move from initial trust to evaluative judgment and then to subsequent strategies such as integration, revision, or abandonment. The present study addresses this gap by investigating that process in the context of upper secondary science learning.

## Methods

### Research design

This study employed a constructivist grounded theory design to investigate how upper secondary students engage with generative artificial intelligence (GenAI) outputs in science learning. A grounded theory approach was appropriate because the study sought to explain an under-theorized process—namely, how students move from initial exposure to AI-generated content toward evaluation, revision, selective integration, or abandonment—rather than to test predefined variables or hypotheses. The constructivist variant was particularly suitable because it treats meanings and categories as co-constructed through the interaction between participants’ accounts and the researchers’ interpretive analysis, while retaining the core grounded theory emphasis on iterative coding, constant comparison, and theory development from data (Charmaz, 2014; Corbin and Strauss, 2015).

The study was designed as a qualitative, interview-based inquiry focusing on students’ authentic experiences of GenAI use in disciplinary learning rather than experimentally scaffolded use. The research questions provided broad sensitizing concerns for the inquiry, directing attention to how students began using GenAI, how they judged AI-generated outputs, and how they responded to those outputs in concrete science-learning situations. Because these concerns involved unfolding judgment and decision processes, a process-sensitive design was necessary.

### Research context and participants

The study was conducted in a major city in Southwest China. Participants were recruited from two upper secondary schools: one provincial key high school and one municipal non-key general high school. This two-site design was used to capture variation in academic demands, classroom expectations, and school-level learning cultures. In the Chinese upper secondary context, these institutional differences are consequential because they shape task stakes, teacher accountability norms, and students’ everyday study strategies.

A total of 21 students aged 15–18 participated in the study (*M* = 16.5). The sample comprised 11 male and 10 female students, including 7 students in Grade 10, 8 in Grade 11, and 6 in Grade 12. Eleven participants were recruited from the key high school and 10 from the non-key high school. To ensure that all participants could provide experience-based accounts rather than speculative opinions, inclusion criteria required at least 3 months of prior GenAI use for science learning tasks, such as concept explanation, problem solving, exam review, and laboratory report support.

Sampling proceeded in two stages. The study began with purposive variation sampling across grade level, school type, and self-reported frequency of GenAI use. As analysis progressed, subsequent participants were selected theoretically to elaborate emerging categories, examine variation in engagement patterns, and test relationships among categories. The final interviews were used to confirm that no substantively new categories or major relationships were emerging. This combination of initial purposive variation and subsequent theoretical sampling strengthened the analytic fit between the data and the emerging model (Charmaz, 2014; Corbin and Strauss, 2015). Table 1 summarizes the participant profile.

**Table 1:** Demographic and basic characteristics of participants.

Characteristic	Category	*n*
Total participants		21
Gender	Male	11
	Female	10
Grade	Grade 10	7
	Grade 11	8
	Grade 12	6
School type	Provincial key high school	11
	Municipal non-key high school	10
Age	Range	15–18
	Mean	16.5
Inclusion criterion	Prior GenAI use for science learning	≥3 months

### Data collection

Data were collected through one-to-one semi-structured interviews conducted between April and June 2025 via a secure online video-conferencing platform. Interviews were conducted in Mandarin Chinese to enable participants to describe their experiences with precision and nuance. Each interview lasted between 45 and 75 min, with an average duration of 58 min.

The interview guide was developed to elicit process-based narratives of students’ actual GenAI use in science learning rather than abstract attitudes toward AI. As shown in Table 2, the interview guide was organized around broad experiential domains, including students’ prior use history, the situations in which they chose to use GenAI, their first reactions to AI-generated output, how they checked and judged that output, what they did afterwards, and what contextual conditions shaped those decisions. These domains were suggested by the research questions at a broad level, but they were not treated as predetermined analytic categories. Instead, they were used to support narrative recall and grounded-theory-style probing during the interviews. Participants were repeatedly encouraged to describe recent and concrete science-learning episodes so that the interviews would capture actual decision sequences, checking practices, and follow-up actions rather than abstract beliefs or normatively desirable claims.

**Table 2:** Development of the semi-structured interview guide.

Interview domain	Purpose in data collection	Example of early draft question	Example of revised question after expert review and pilot interviews
Background and use history	To establish participants’ prior GenAI exposure, typical science-learning uses, and frequency of use	What AI tools do you usually use for learning?	What GenAI tools have you used for science learning in the past 3 months, and for what kinds of tasks (e.g., concept explanation, problem solving, exam review, laboratory reports)?
Starting points and typical situations of GenAI use	To elicit recent, concrete episodes in which students chose to use GenAI in science learning.	Do you trust GenAI in science learning?	Can you describe a recent science-learning task in which you used GenAI? Why did you decide to use it in that situation rather than another resource?
First responses to AI-generated output	To explore students’ immediate reactions to the first answer they received, including perceived usefulness, plausibility, and initial trust or doubt	What do you think of GenAI answers?	When you first saw the response, what was your reaction? What made it seem useful, convincing, incomplete, or questionable to you?
Checking and judging AI outputs	To investigate how students evaluated the reliability, correctness, and task-fit of GenAI-generated content	How do you verify whether GenAI is correct?	After reading the answer, what did you do next? Did you compare it with a textbook, class notes, your teacher’s explanation, or another source? How did you decide whether the answer was reliable enough to use?
Follow-up actions after using GenAI	To examine what students actually did with the output after judging it, including revising, integrating, rewriting, or discarding it	How do you use AI answers in your homework?	What did you finally do with the response? Did you use it directly, revise it, combine it with other materials, rewrite it in your own words, or decide not to use it?
Contextual influences on use	To capture situational factors shaping students’ decisions, such as task stakes, deadlines, prior knowledge, and teacher norms	Does your teacher allow AI use?	Did the type of task, the deadline, your confidence in the topic, or your teacher’s expectations affect whether you trusted or checked the answer? Can you give an example?
Closing reflection	To invite clarification, additional examples, and reflections not covered by earlier questions	Do you have anything else to add?	Is there any experience of using GenAI in science learning that we have not discussed but that you think is important for understanding how students actually use it?

The interview guide was organized around broad experiential domains suggested by the research questions, but these domains were used to elicit narrative accounts rather than to impose predetermined analytic categories. The wording and sequencing of questions were refined after expert review and pilot interviews in order to generate more concrete, episode-based responses.

The interview guide was refined in three stages. First, an initial draft of the interview guide was reviewed by two science education scholars with expertise in qualitative research and scientific literacy, who provided feedback on disciplinary relevance, wording clarity, and probing depth. Second, pilot interviews were conducted with three upper secondary students who were not included in the final sample. These pilots showed that broad attitudinal questions often generated generalized answers, whereas recent, task-specific prompts produced richer accounts of decision sequences. Several questions were therefore revised to encourage participants to reconstruct concrete episodes of GenAI use, as illustrated in Table 2. Third, the interview guide remained open to iterative adjustment during data collection in response to early analytic insights, consistent with grounded theory practice. For example, additional follow-up probes were introduced when early interviews suggested that task stakes, prior knowledge, time pressure, and teacher norms were shaping students’ trust judgments and checking practices.

To reduce the risk of socially desirable self-presentation, participants were repeatedly asked to recount what they had actually asked GenAI, what the system returned, what they checked or did not check, and what they ultimately used, revised, or abandoned. The interview framing emphasized decision-making experiences rather than moral evaluation, which helped participants discuss both careful and less careful forms of GenAI use.

All interviews were audio-recorded with consent and transcribed verbatim by a professional transcription service. During transcription, identifying information was removed and participants were assigned pseudonymous identifiers (S01–S21). The cleaned transcripts were then imported into NVivo 14 to support data management, coding, memo-writing, and cross-case comparison.

### Data analysis

Data analysis proceeded concurrently with data collection and followed the grounded theory sequence of initial/open coding, axial coding, and selective coding, together with constant comparison across interviews, incidents, and emerging categories (Charmaz, 2014; Corbin and Strauss, 2015). This concurrent design allowed early analytic insights to inform later sampling and interviewing.

In the first stage, transcripts were coded line by line to identify actions, judgments, rationales, and contextual conditions in participants’ accounts. Coding stayed as close as possible to participants’ own wording in the early phase in order to avoid imposing an external conceptual grid too quickly. The author and a trained research assistant independently coded an initial subset of transcripts and discussed discrepancies until a shared interpretive understanding and provisional codebook were established. This early comparison functioned as an analytic calibration exercise rather than a positivist reliability test.

In the second stage, conceptually related codes were grouped into higher-order categories by examining conditions, interactions, and consequences. Through this process, three main categories were developed: interaction initiation and initial acceptance, cognitive evaluation and epistemic judgment, and behavioral decision-making and content reconstruction. These categories were generated through iterative coding and constant comparison rather than used as an *a priori* coding template. They are later used to organize the findings for reporting purposes, but they do not imply a fixed sequence that every participant followed in the same way. Contextual moderators, such as task epistemic demand, time pressure, classroom teacher norms, and prior scientific knowledge, were identified during this phase.

In the final stage, selective coding was used to integrate the categories into an explanatory model of students’ engagement with GenAI in science learning. Supplementary Appendix B presents the condensed coding framework derived from this final analytic structure. Its purpose is to show the final analytic architecture that emerged through iterative coding, constant comparison, and theoretical integration.

Theoretical saturation was treated as a conceptual rather than purely numerical criterion. After 17 interviews, no new categories or major relationships were emerging; the final four interviews were therefore used primarily to test category robustness and confirm saturation.

After selective coding, a case-by-category matrix was prepared to document which participants articulated each subcategory and how subcategories co-occurred across cases. The revised Findings section draws on this matrix to report descriptive counts and to summarize selected cross-category pathways in the text.

### Trustworthiness and ethical considerations

To enhance trustworthiness, the study adopted several established qualitative strategies. First, constant comparison was applied throughout the analysis, so that new incidents were repeatedly checked against earlier codes and categories. Second, analytic memos were maintained throughout data collection and analysis to document category development, emerging questions, and theoretical decisions. Third, peer debriefing was conducted at key analytic stages with two external science education scholars, which helped refine category boundaries and challenge premature interpretations. Fourth, negative cases were actively sought and incorporated to sharpen the explanatory scope and limits of the emerging model. Finally, a reflexive journal and audit trail were maintained to document interview refinements, coding decisions, category revisions, and the researchers’ evolving interpretations. Together, these procedures supported credibility, dependability, and transparency in line with qualitative inquiry principles (Lincoln and Guba, 1985).

The study received approval from the author’s institutional review board. Written informed consent was obtained from all participants, and parental or guardian consent was obtained for minors. Participants were informed of the voluntary nature of participation, their right to withdraw, and the confidentiality arrangements surrounding audio files and transcripts. To minimize risk, interview questions focused on decision-making experiences rather than moral judgment of students’ behavior, and all reported data were anonymized before analysis.

## Findings

### Overview of the core pattern

The analysis suggests that upper secondary students’ engagement with GenAI in science learning is best understood as a process of movement from initial trust to increasingly critical reconstruction. The main categories reported below are analytic groupings used to present the dominant pattern rather than fixed stages. Students often described a recurring sequence in which they first turned to GenAI for practical reasons, then evaluated the quality and suitability of its outputs, and finally decided how those outputs should be used, revised, or rejected. Across interviews, this process was shaped by task stakes, time pressure, teacher norms, and students’ prior scientific knowledge. These factors did not determine behavior in a fixed way, but they consistently influenced how much trust students granted at the outset, how carefully they checked AI-generated content, and whether they ultimately incorporated it into their own work. Two participants were skeptical from the outset and did not pass through an initial-trust stage at all, while others moved back and forth between checking, reconstruction, and abandonment.

### Main category 1: interaction initiation and initial acceptance

This category captures how students began using GenAI in science learning and what shaped their initial orientation toward its responses. In most accounts, initial engagement was driven by a combination of instrumental efficiency and surface-level trust. At the same time, this initial acceptance was not stable across tasks. Students repeatedly emphasized that they trusted GenAI differently depending on the type of science task, the stakes involved, and the classroom norms surrounding AI use.

#### Efficiency-driven instrumental adoption (*n* = 19/21)

For many students, the immediate reason for turning to GenAI was practical rather than epistemic. They used it to save time, reduce workload, and cope with the pressure of homework, revision, and multiple competing tasks. In these situations, GenAI was often treated as a fast-response tool that could provide ready-made explanations, summaries, or solution paths more quickly than textbooks or teachers.

As one student explained, “There’s just too much physics homework every night. When I can’t finish the problems before the deadline, I just feed the question into Deepseek, and it instantly gives me the full steps and answer. It’s so convenient, it saves me at least an hour every day” (S03). Another participant described using GenAI during exam preparation: “Near final exams, I have to review six subjects at once. I use GenAI to help me summarize the key points of each chapter and draft an outline for my review. It saves me a huge amount of time that I can spend on memorizing and practicing” (S07).

These accounts show that initial GenAI use was often task-completion oriented. In such situations, efficiency concerns typically came before careful evaluation. When the primary goal was to finish work quickly, students were more likely to accept outputs provisionally and only question them later if something seemed obviously wrong.

#### Initial trust under the halo of technical authority (*n* = 16/21)

A second pattern was students’ tendency to trust GenAI because of the way its responses looked and sounded. Participants often described AI-generated outputs as fluent, well-organized, technical, and confident. For students dealing with unfamiliar science topics, these features often created the impression that the answer was probably reliable, even before any real checking had taken place.

One participant stated, “When I read the response, it looks very professional, full of technical terms that I barely understand, and it’s written in a very confident way. My first reaction was to trust it, because it looks like it knows what it’s talking about, way more than I do” (S01). A similar point appeared in another interview: “When I’m learning a new physics concept that I don’t understand at all, I’ll ask GenAI to explain it. If the explanation is fluent and uses the right terms, I’ll just accept it, because I don’t have any prior knowledge to tell if it’s wrong” (S20).

This kind of initial trust was typically heuristic rather than evidence-based. Students were not claiming that they had confirmed the output’s accuracy; rather, they were responding to the textual cues of authority. However, this tendency was weaker among students who felt more confident in their own science knowledge, suggesting that prior knowledge shaped not only later evaluation but also the first encounter with the response.

#### Task-context dependency of initial acceptance (*n* = 20/21)

Although many students described a tendency toward initial acceptance, they also made clear that trust was highly dependent on task context. GenAI was trusted more readily for low-stakes tasks such as brainstorming, summarizing, outlining, or generating initial ideas, but much less for tasks requiring precise reasoning, experimental design, or exam-relevant accuracy.

As one student put it, “For writing a general argumentative essay about science, its ideas can be quite fresh and useful, I’ll trust it more for that. But if it’s designing an experiment to verify Newton’s second law, I wouldn’t dare fully trust it at all” (S05). Another participant drew a sharp distinction between exam preparation and routine homework: “For the college entrance exam practice problems, I never trust GenAI’s answers directly. Those are high stakes, if I learn the wrong method, I’ll lose points. But for casual homework the teacher won’t grade carefully, I’ll trust it more” (S09).

Teacher norms also shaped this initial stance. Several students explained that they adjusted their GenAI use depending on whether teachers allowed bounded use, discouraged direct copying, or banned GenAI entirely. Initial acceptance was therefore not a fixed personal trait, but a situational orientation shaped by the task and learning environment.

### Main category 2: cognitive evaluation and epistemic judgment

This category concerns how students judged whether GenAI-generated science content was reliable and usable. Across interviews, evaluation was not described as a single act but as a layered process. Students typically began with quick internal checks, and if uncertainty remained—especially in high-stakes situations—they moved to external verification. Over time, repeated encounters with GenAI errors also fostered stronger awareness of its limitations.

#### Internal consistency checking based on prior knowledge (*n* = 21/21)

The most immediate evaluation strategy was to compare the AI response against what students already knew from textbooks, classroom teaching, or basic scientific reasoning. When the answer conflicted with familiar knowledge or seemed logically inconsistent, students reported immediate doubt.

One participant recalled, “It explained photosynthesis, but it reversed the light-dependent and light-independent reactions, and mixed up the inputs and outputs. I could tell immediately it was wrong, because I memorized that entire process from the textbook backwards and forwards” (S02). Another student said, “If it says something that goes against what my teacher taught us in class, I’ll immediately doubt it. My teacher has been teaching chemistry for 20 years, I trust her more than the AI” (S10).

This kind of checking functioned as a first filter. It was fast and low-cost, but its effectiveness depended heavily on what the student already knew. Those with stronger prior knowledge described noticing more subtle problems, whereas those with weaker backgrounds often said that they could only detect obvious errors.

#### Cross-validation through external information sources (*n* = 17/21)

When students could not judge the answer internally, or when the task carried higher stakes, many turned to external sources for verification. These sources were usually used in a hierarchy: textbooks and official materials first, then reputable online resources, and finally teachers or high-achieving peers when confirmation was especially important.

As S04 explained, “If it’s a concept I don’t know well, I can’t tell if it’s wrong just by reading it. So I’ll check the textbook first, to see what the official explanation is. If the AI’s answer matches the textbook, then I’ll trust it. If not, I’ll go with the textbook.” In higher-stakes situations, human confirmation became particularly important. S17 noted, “If it’s an important knowledge point that will be on the college entrance exam, I’ll never just trust the AI. I’ll write down the explanation it gave me, and ask my teacher the next day to confirm if it’s correct.”

These accounts show that many students treated AI-generated output as provisional material that still required confirmation. At the same time, external checking was effortful and was not carried out consistently across situations. Several participants explicitly noted that they were more likely to suspend or skip external checking when deadlines were tight.

As S09 put it, “If the assignment is due the next day, I simply don’t have time to check if what the AI said is correct. I’ll just tweak what it generated a bit and submit it. Anyway, teachers don’t check every single point in detail. If there are any mistakes, I can correct them when the teacher points them out in the grading.” Similarly, S21 shared, “When rushing to meet a deadline, I’ll use the AI’s answers first to finish and submit the assignment on time. I’ll put a question mark next to those parts to remind myself to go back and check the textbook or other resources when this busy period is over and I have free time. But more often than not, I forget about it once I’m done with the rush, and never end up verifying them.” These cases suggest that awareness of the need for verification did not always translate into actual checking behavior. Under deadline pressure, epistemic caution was often overridden by the practical need to complete and submit tasks on time.

#### Metacognitive awareness of GenAI limitations (*n* = 18/21)

A further shift in evaluation occurred when students developed explicit awareness that GenAI could sound convincing while still being wrong. This awareness was usually not theoretical; it emerged from lived experience of failure, such as wrong answers, inconsistent outputs, or poor performance after relying too heavily on AI-generated responses.

One student described a turning point: “At first I thought it was like a super smart teacher that never made mistakes. But then I used it to solve a physics problem, and it gave me the wrong answer, and I got a bad grade on the homework. That’s when I realized it’s just a knowledgeable parrot that sometimes just makes up stories” (S03). Another participant reflected on repeated inconsistency: “I’ve seen it give three different answers to the same physics problem when I ask it three times in a row. That’s when I realized it doesn’t actually ‘understand’ the problem, it’s just generating plausible-sounding text. Now I never trust it to give me a final answer” (S21).

This awareness marked a transition from default acceptance to more cautious and deliberate use. Once students had experienced AI error directly, they were less likely to treat GenAI as an authority and more likely to approach it as a fallible tool.

### Main category 3: behavioral decision-making and content reconstruction

This category captures what students actually did after evaluating GenAI outputs. The data suggest three recurring responses: they refined the interaction through better prompting, transformed the output through selective reconstruction, or abandoned GenAI when the cost or risk became too high.

#### Iterative prompt refinement (*n* = 15/21)

Many students reported that effective use of GenAI required learning how to ask better questions. Instead of accepting shallow first responses, they began to refine prompts by specifying level, scope, context, and format.

As S11 explained, “At first I just asked ‘Why is the sky blue?’ and the answer was really shallow. Later I learned to be specific: ‘Explain why the sky is blue using Rayleigh scattering, at high school physics level, with no university math, and step-by-step formula explanation.’ Then the answer was actually useful for my class.” A similar pattern appeared in more extended interactions. S08 described how prompt refinement became an iterative process of adjustment: “For my biology experiment design, I didn’t just ask it to design the experiment once. I had a whole back-and-forth with it. First I asked for a draft, then I told it to add a control group, then to adjust the variables to match the equipment we have in our school lab, then to fix the data analysis method. By the end, the design was actually usable, because I kept refining the prompt to fit my needs.”

These accounts suggest that some students moved from passive reception to active management of the interaction itself. At the same time, this practice was unevenly distributed across the sample: some students described iterative prompting as routine, whereas others remained closer to one-shot querying.

#### Content integration and reconstruction (*n* = 19/21)

A second and more substantial response involved transforming GenAI output into work the students considered their own. This often included selecting useful points, reorganizing structure, paraphrasing language, and integrating textbook evidence or personal reasoning.

S07 described this process as follows: “I never copy directly. I read the whole response, pick out the key points and logical structure that make sense, then mix those with my own ideas and evidence from the textbook, and restate everything in my own words. By the end, it’s my work, not the AI’s.” S12 expressed a similar idea through a vivid metaphor: “I have a metaphor for this: the AI’s paragraphs are like bricks. I don’t just take the whole house the AI built and put my name on it. I pick out the bricks I need—the good ideas, the correct facts, the useful structure. Then I build my own beams and pillars: my original viewpoints, my own reasoning, the evidence from my experiments and the textbook. Then I put the whole house (the assignment) together myself. The AI gives me materials, but I’m the architect.” In these cases, GenAI served as raw material rather than a final answer.

Other students adopted a more superficial approach to revision. As S16 admitted, “If it’s a simple homework assignment or a low-stakes exercise, I won’t put too much effort into changing it. I mostly just rephrase the sentences the AI wrote, adjust the word order, and replace any overly formal or awkward words with ones I would normally use. The content and structure are basically what the AI gave me; only the language is mine.”

The depth of reconstruction therefore varied. For some, revision meant mostly rewording. For others, it involved deeper reorganization and evidence-based rewriting. Taken together, these cases show that students’ reconstruction ranged from surface-level paraphrase to more substantial evidence-based re-authoring.

#### Strategic abandonment and substitution (*n* = 16/21)

The third response was to stop using GenAI for a specific task and turn instead to self-solving, peers, teachers, or traditional resources. Students described doing this when outputs were repeatedly inconsistent, unrealistic, or too costly to repair.

One participant explained, “I asked it to solve a hard calculus derivative problem for physics, and it gave two wrong answers with made-up reasoning. I realized it can’t handle this kind of multi-step creative problem-solving. So I closed it, worked it out myself step by step, and asked my teacher to check it. It was faster that way than fixing the AI’s mistakes” (S09). Another student reported abandoning AI-generated experimental advice because it did not fit the school laboratory context: after repeated unrealistic designs, the student “gave up, and designed the experiment myself with my lab partner, using the equipment we actually have” (S13).

Importantly, abandonment did not usually mean rejecting GenAI completely. Instead, it reflected a judgment that, for that task, the tool was not worth the epistemic risk or revision cost.

Taken together, the findings show that upper secondary students’ engagement with GenAI in science learning was neither uniformly trusting nor uniformly critical. It typically began with practical efficiency and authority-based first impressions, but then became differentiated through evaluation, verification, and strategic response. Across cases, students rarely remained in a single stance toward GenAI. Many began with efficiency-oriented or authority-based acceptance, but later differentiated their responses through checking, corroboration, and accumulated experience of error. Depending on task stakes and revision costs, they then refined prompts, reconstructed outputs, or abandoned GenAI for particular tasks. Taken together, these patterns are consistent with understanding students’ GenAI use as a form of regulated engagement.

Cross-case comparison also showed patterned transitions across the three main categories. Of the 16 students who reported initial trust under the halo of technical authority, all 16 later described heightened awareness of GenAI limitations after encountering inaccuracies or inconsistencies. Within this initial-trust group, 14 students subsequently reported prompt refinement and/or selective reconstruction, and 14 reported strategic abandonment in at least some high-risk tasks. These two response patterns overlapped but were not identical: 12 students reported both patterns, 2 reported only prompt refinement and/or selective reconstruction, and 2 reported only strategic abandonment. Two additional students were skeptical from the outset and therefore did not pass through the initial-trust pathway, although they also articulated awareness of GenAI limitations.

## Discussion

### Reframing students’ GenAI use as regulated epistemic engagement

The central contribution of this study is to show that upper secondary students’ engagement with GenAI in science learning is better understood as a process of regulated epistemic engagement than as a simple matter of adoption, acceptance, or frequency of use. Much of the existing literature has examined whether students use ChatGPT, how useful they perceive it to be, or what concerns they report about it (Acosta-Enriquez et al., 2024; Baek et al., 2024; Polyportis and Pahos, 2025; Stöhr et al., 2024). Those studies have established that GenAI is now embedded in students’ learning lives, but they leave open a more educationally consequential question: what students actually do after receiving AI-generated content. The present findings suggest that the critical issue is not use per se, but how learners regulate trust, evaluate output quality, and decide whether AI-generated content should be incorporated, revised, or rejected.

This reframing shifts attention to what students did with AI-generated content after receiving it, including whether they verified it, revised it, integrated it, or abandoned it. In the present study, students did not simply “use AI” or “avoid AI.” They moved through a sequence in which practical attraction and authority cues often initiated engagement, while later judgment depended on prior knowledge, corroboration, and task demands. This pattern extends recent work suggesting that students’ reliance on GenAI is heterogeneous and situational rather than uniform (Stojanov et al., 2024). It also suggests that school-based GenAI use is best understood as an ongoing regulatory process in which learners continuously negotiate the value and limits of machine-generated content.

This interpretation also complements self-regulated learning research. Zimmerman (2000) conceptualized learning as cyclical activity involving forethought, monitoring, control, and reflection. The findings show that GenAI became part of that cycle itself. Students planned when to use it, monitored the adequacy of its responses, adjusted their prompts, and decided whether the resulting output could support their goals. In practice, GenAI often redistributed cognitive work instead of removing it: students may have saved time at one stage, but they still needed to judge output quality, verify claims, and decide how to use the material responsibly.

### Trust calibration is task-contingent, not a stable learner trait

A second contribution of the study is to show that students’ trust in GenAI was task-contingent and disciplinarily calibrated, not a stable individual disposition. Research on trust in automation and AI has long argued that the educationally desirable outcome is neither blind trust nor blanket distrust, but appropriate reliance aligned with system capability and situational risk (Glikson and Woolley, 2020; Lee and See, 2004). The present findings support that general proposition, but they also refine it in an important way. In the science learning context, students did not apply one general “trust level” to GenAI. Instead, they adjusted their acceptance thresholds depending on the epistemic demand and stakes of the task.

Students were often willing to accept GenAI more readily for brainstorming, summarizing, or low-stakes organizational support, but became far more cautious when tasks required precise reasoning, experimental design, or exam-relevant accuracy. This distinction suggests that students’ trust judgments in science learning were shaped by disciplinary demands for evidentiary adequacy and conceptual precision as well as by general perceptions of AI usefulness. Science tasks often require evidence, precision, and defensible reasoning. Students therefore calibrated trust partly in relation to disciplinary expectations, not just to general usefulness.

This finding also helps explain why some adoption-focused studies may overestimate the educational value of reported GenAI use. A student may express high willingness to use ChatGPT while simultaneously distrusting it for exactly those science tasks that matter most for disciplinary learning. In that respect, the present findings add nuance to research showing positive effects of ChatGPT on engagement or performance under scaffolded conditions (Heung and Chiu, 2025; Lo et al., 2024; Ng et al., 2024a). Such benefits may well be real, but they are unlikely to depend on use alone. They depend on whether students can discriminate between tasks for which GenAI is educationally serviceable and tasks for which its outputs require intensive verification or should be avoided altogether.

The findings further indicate that prior knowledge is central to this calibration process. Students with stronger science backgrounds were better able to detect subtle inconsistencies and to resist the rhetorical pull of technically fluent but weak explanations. This aligns with research showing that epistemic beliefs and metacognitive accuracy shape how students integrate GenAI-generated content into academic work (Urban et al., 2025). It also suggests a potentially troubling inequality: the students who most need support may be those least equipped to judge the reliability of the support they receive.

### From verification to reconstruction: the educational value of active appropriation

The findings suggest that reconstruction was the most demanding form of post-evaluative engagement reported by students. When students selected usable content, reorganized it, added textbook evidence, and re-expressed it through their own reasoning, they retained greater control over the final work than when they only checked whether an AI response was correct. In the present data, reconstruction required more than error detection: it involved deciding which parts of the output could be kept, which needed revision, and how the final explanation should be justified in disciplinary terms. This distinction helps clarify what responsible GenAI use looked like in these science-learning contexts. Productive use did not depend on skepticism alone, but on whether students could transform AI-generated material into accountable work that met the epistemic demands of the task.

This insight also extends AI literacy research. Existing frameworks have productively identified cognitive, behavioral, affective, and ethical dimensions of AI literacy (Ng et al., 2024b), yet they often remain broad at the level of competence domains. The present study adds process detail by showing how literacy was enacted in concrete moments of science learning. Students demonstrated this literacy by corroborating claims, refining prompts, adding evidence, and deciding when the cost of repair exceeded the value of using the tool. In this sense, critical reconstruction can be understood as a situated form of AI literacy within disciplinary practice.

These findings also matter for science education more broadly. Science literacy has long been framed as the capacity to engage with evidence, expertise, and scientific claims rather than simply accumulate facts (Bromme and Goldman, 2014; Feinstein, 2011). GenAI intensifies this demand because it produces text that often looks epistemically robust while obscuring the basis on which claims are generated. Recent work in science education has therefore called for closer attention to the epistemic implications of AI-mediated knowledge practices (Cheung et al., 2025; Erduran and Levrini, 2024). The present findings suggest that, in AI-rich environments, science literacy should include at least three linked capacities: calibrating trust in AI-generated claims, corroborating outputs against authoritative sources and disciplinary expectations, and reconstructing AI-generated content into evidence-aligned scientific understanding.

### Implications for science teaching in AI-mediated classrooms

The practical implications of these findings are less about whether schools should permit GenAI and more about what kind of classroom norms and pedagogies make critical use possible. First, the results suggest that simply telling students to “use AI responsibly” is insufficient. Students were much more likely to verify outputs when task stakes were high or when verification was embedded in the work itself. This implies that teachers should treat verification not as a moral injunction, but as a routine part of scientific work. Classroom tasks can be designed to require students to document how they checked AI-generated claims against textbooks, class notes, or experimental evidence.

Second, teachers may need to teach reconstruction explicitly. Several students described forms of use that remained relatively superficial, consisting largely of paraphrasing or surface modification. If schools want GenAI to support learning rather than shortcut it, students need instruction in how to select, reorganize, justify, and rewrite AI-generated material so that the final product reflects disciplinary standards and personal understanding. In this respect, process artifacts such as annotated drafts, comparison notes, or verification records may become as important as the final answer.

Third, teacher norms matter. The findings suggest that blanket bans do not remove GenAI from students’ learning environments; instead, they may push use into less visible and less accountable forms. By contrast, bounded and explicit guidance appears more likely to support critical engagement. This does not mean unrestricted permissiveness. Rather, it suggests that science classrooms need norms distinguishing acceptable support functions, such as idea generation or initial explanation, from uses that bypass reasoning or evidence.

### Limitations and directions for future research

This study should be interpreted with consideration of several limitations. The sample was restricted to 21 students from two upper secondary schools in a single city in Southwest China, which renders the findings analytically informative but not statistically generalisable and highlights the need for further research across more diverse school systems, regions, and cultural settings. The study relied on interview-based self-report data; while the interviews elicited detailed narratives, students may have underreported copying or overstated their verification and reconstruction behaviors, so future research should triangulate interview data with behavioral traces such as prompt histories, assignment drafts, teacher feedback, or screen recordings. As a qualitative process study, this research identifies patterns of engagement rather than measuring their prevalence or causal relationships, and future work can build on these findings to develop instruments for assessing task-contingent trust calibration, verification practices, and reconstruction strategies at scale. The study also focused primarily on students’ individual engagements with GenAI, suggesting that future research should examine how classroom interventions, peer culture, parental expectations, and platform design interact with disciplinary demands to shape students’ AI use over time.

## Conclusion

This study examined how upper secondary students engage with GenAI in science learning and found that their engagement followed a process from initial trust to critical reconstruction. Students often turned to GenAI for efficiency, clarification, and rapid support, and many initially responded to fluent, technical outputs with provisional trust. Their later actions were shaped by a more complex process of judgment: they checked outputs against prior knowledge, corroborated them with textbooks or teachers when necessary, and then decided whether to refine, reconstruct, or abandon the generated content.

The study makes three related contributions to current scholarship. First, it directs attention away from broad adoption measures and toward the process through which students evaluated and acted on AI-generated scientific content. Second, it shows that students’ trust judgments were shaped by task stakes, epistemic demand, and prior knowledge. Third, it highlights reconstruction as an important form of engagement, because students often learned more productively when they reorganized and re-authored GenAI output than when they accepted it with minimal revision.

These findings have important implications for science education. As GenAI becomes increasingly embedded in students’ learning environments, the central issue is how schools can help students use it while preserving epistemic responsibility. Science classrooms therefore need clear norms for AI use and pedagogies that cultivate verification, discernment, and accountable reconstruction. In AI-mediated learning environments, supporting these capacities is likely to become an essential part of scientific literacy education.

## Data Availability

The original contributions presented in the study are included in the article/Supplementary material, further inquiries can be directed to the corresponding author.
