# Supplementary material for: From initial trust to critical reconstruction: upper secondary students’ engagement with generative AI in science learning
Source: Front Psychol. 2026 May 22;17:1830646. doi: 10.3389/fpsyg.2026.1830646 (PMC13236895; doi:10.3389/fpsyg.2026.1830646)
Supplement: Supplementary file 1 [file Supplementary_file_1.docx]

# Appendix A. Semi-Structured Interview Protocol for Upper Secondary Students

**Purpose.** The interview protocol was designed to elicit process-based accounts of students’ authentic experiences of using generative AI in science learning. The full protocol was aligned with the three research questions and organised into four substantive modules plus opening and closing segments.

**Administration notes.** Interviews were conducted in Mandarin Chinese, audio-recorded with permission, and used recent, concrete examples rather than general opinions. Probes were flexibly adapted in line with grounded theory interviewing principles.

| **Module** | **Focus** | **Illustrative main questions / probes** |
| --- | --- | --- |
| Opening & background | Consent, anonymity, prior GenAI use, frequency, and science-task types. | Opening consent script; grade level; tools used; length and frequency of GenAI use; typical science learning tasks. |
| Module 1 | Interaction initiation and initial acceptance (RQ1). | Recent science task using GenAI; reasons for selecting GenAI over textbooks/teachers/peers; first reaction to the response; task conditions that increase trust or caution. |
| Module 2 | Cognitive evaluation of AI-generated scientific content (RQ2). | How students check reliability and correctness; how they identify errors; what sources they use to cross-validate; perceived limitations of GenAI in science learning. |
| Module 3 | Behavioural decision-making and content reconstruction (RQ3). | What students do after evaluation; prompt refinement; integration or rewriting of GenAI outputs; strategic abandonment and substitution. |
| Module 4 | Contextual factors and supplementary exploration. | Teacher norms and classroom policies; peer influence; additional experiences relevant to GenAI use in science learning. |
| Closing | Opportunity for clarification and participant questions. | Invitation to add further comments; optional request to receive a summary of findings. |

**Core background questions.** Grade level; GenAI tools used for science learning; duration of use; frequency of use; and common science-task types (e.g., concept explanation, problem solving, laboratory reports, brainstorming, or exam review).

# Appendix B. Condensed Coding Framework for Grounded Theory Analysis

**Purpose.** This appendix presents the final analytic framework derived from iterative open, axial, and selective coding. It is intentionally condensed for reporting purposes and should be read as a grounded theory coding framework rather than a thematic analysis manual.

Analytic note. The three main categories and nine subcategories emerged through constant comparison across interviews. Contextual moderators identified during analysis included task epistemic demand, time pressure, classroom teacher norms, and prior scientific knowledge.

| **Main category** | **Subcategory** | **Operational focus** | **RQ** |
| --- | --- | --- | --- |
| Interaction initiation and initial acceptance | Efficiency-driven instrumental adoption | Students turn to GenAI primarily to save time, reduce workload, or accelerate task completion in science learning. | RQ1 |
| Interaction initiation and initial acceptance | Initial trust under the halo of technical authority | Students initially accept outputs because fluent language, technical vocabulary, and confident tone cue perceived expertise. | RQ1 |
| Interaction initiation and initial acceptance | Task-context dependency of initial acceptance | Initial trust varies with task stakes, epistemic demand, teacher norms, and topic familiarity. | RQ1 |
| Cognitive evaluation and epistemic judgement | Internal consistency checking based on prior knowledge | Students compare GenAI responses with textbook memory, prior scientific knowledge, and logical coherence. | RQ2 |
| Cognitive evaluation and epistemic judgement | Cross-validation through external information sources | When internal checking is insufficient, students corroborate GenAI outputs with textbooks, curriculum materials, teachers, or other authoritative sources. | RQ2 |
| Cognitive evaluation and epistemic judgement | Metacognitive awareness of GenAI limitations | Students recognise hallucinations, inconsistency, and the system’s lack of genuine scientific understanding, usually after encountering errors. | RQ2 |
| Behavioural decision-making and content reconstruction | Iterative prompt refinement | Students refine prompts and engage in follow-up questioning to improve output relevance, accuracy, and task fit. | RQ3 |
| Behavioural decision-making and content reconstruction | Content integration and reconstruction | Students selectively integrate, paraphrase, reorganise, and reconstruct GenAI outputs with their own reasoning and evidence. | RQ3 |
| Behavioural decision-making and content reconstruction | Strategic abandonment and substitution | Students stop using GenAI for a task when epistemic risk or revision cost becomes too high and shift to textbooks, teachers, peers, or self-solving. | RQ3 |

**Interpretive caution.** The coding framework summarises the dominant process identified in the data. It is intended to support transparency of analysis rather than to function as a fixed checklist detached from the interview context and analytic memos.
